# Estimates of subnational health trends in Kenya

**DOI:** 10.1016/S2214-109X(18)30516-3

**Published:** 2019-01-01

**Authors:** Emelda A Okiro

**Affiliations:** Population Health Unit, KEMRI Wellcome Trust Research Programme, PO Box 43640-00100, Nairobi, Kenya

The Millennium Development Goals stimulated improved use of health metrics and disease burden estimates. Progress in most countries has been monitored at national levels, yet differences in the rates of change in mortality exist within countries, with considerable geographic inequalities with respect to the distribution and use of health resources. Knowing where and how to improve national health and health services is limited by a paucity of adequate data at subnational levels.^1^ Consequently, efforts to track progress subnationally rely on modelling. In *The Lancet Global Health*, Tom Achoki and colleagues^2^ report an evaluation of mortality, morbidity, and risk factors, adapting Global Burden of Diseases, Injuries, and Risk Factors Study (GBD) methodology at subnational units (counties), to produce a report on health trends in Kenya from 1990 to 2016.

Kenya provides a unique national context. A new constitution with devolved governance was adopted in 2013 in which administration and health planning functions were transferred to 47 county governments. More recently, the Government of Kenya—through a declaration of the President—announced a new plan, the Big Four, which will guide the development agenda of the country in the period 2018–22, with priorities being universal healthcare coverage, food security and nutrition, enhancing manufacturing, and affordable housing. Other initiatives include the First Lady’s Beyond Zero initiative, which seeks to ensure that no woman dies while giving birth. Additionally, Kenya is at the forefront of the Health Data Collaborative, supporting global efforts to increase accountability and improve quality data for decision making. Improved targeted disease control—with effective and equitable resource allocation—demand a more detailed understanding of subnational risks, intervention coverage, and health outcomes.

Achoki and colleagues^2^ compare trends in two periods, from 1990 to 2006 (providing a historical perspective) and from 2006 to 2016 (reflecting current policies pertinent to policy-making efforts today). Overall mortality in Kenya decreased from 850·3 deaths per 100 000 in 1990 to 579·0 deaths per 100 000 in 2016. Under-5 and maternal mortality showed similar declining trends, with the most substantial drops witnessed post 2006, corresponding to the rollout of free maternity services, new vaccines (eg, pneumococcal conjugate, *Haemophilus influenzae* type b, and rotavirus), scale-up of effective malaria and antiretroviral treatments, and expanded free delivery of bednets and preventive interventions for HIV/AIDS.

Against a background of high infectious disease control, the leading national risk factors in Kenya in 2016 were unsafe water, sanitation, and handwashing; unsafe sex; and malnutrition.^2^ Although there has been an improvement in access to safe drinking water, more than 45% of rural populations do not have access to improved sanitation.^3^ Young people have the highest rates of new HIV infections,^4^ and Achoki and colleagues show that they have high rates of HIV-specific mortality.^2^ Combined with existing initiatives, Kenya needs to intensify promotion of infection prevention and health education, the empowerment of young women,^4^ social and environmental factors, and improved nutrition and food security. A key challenge in achieving universal healthcare coverage—and one that is highlighted by this study—is the rise in the relative contribution of noncommunicable diseases across all counties and the shift in ranking of related risk factors noted in specific counties, particularly those in urban areas.^2^ This has the potential to further constrain an already overwhelmed health system.

The study by Achoki and colleagues reports substantial heterogeneity across counties. There was a 15-year difference between the county with the lowest life expectancy and the county with the highest life expectancy in 2016. Inequality between counties was even more pronounced for maternal mortality, in which a 13-fold difference in maternal mortality was estimated between the best-performing and worst-performing counties. Such disparities are sobering.

However, we need to be cautious about model predictions based on sparse and imperfect data. The GBD project provides a data-quality rating for each country,^5^ with scores ranging from 0 (worst) to 5 (best); Kenya received a rating of 1, suggesting inherent challenges with data quality. Poor-quality data limits the interpretation and validity of such disease burden estimates, yet many policy makers tend to ignore these constraints, with the results of GBD typically assumed to be true without considering their limitations. For the first time ever, the Kenyan Demographic and Health Survey (DHS) 2014^3^ was powered to provide countylevel estimates for specific health indicators; previously, the DHS was unable to provide precision at county levels. The cost of assembling decentralised data requires massive investments; yet, as decentralised health planning becomes the norm across other countries in Africa, in Nigeria and the Democratic Republic of the Congo, we can no longer continue to rely on modelling of incomplete imperfect data, and a move needs to be made towards data collection powered at federal health resource decision-making units.

Separately, data availability was a challenge in Achoki and colleagues’ study, and there are potential issues with model specifications and circularity introduced by using covariates to improve predictions of health indicators. It is rarely the case that data for causes and risk factors of health loss will be available subnationally and in sufficient volume both spatially and temporally; thus, the models might be overfitted or result in covariate-driven metrics for some small areas. This possibility is especially important for areas that show atypical associations between available predictor variables and health indicators. Finally, the temptation to make predictions for periods in which no empirical data are available—particularly with respect to mortality estimates—leads to large uncertainty and sometimes to outputs that are unreliable, which should be recognised. The latest mortality survey data available for Kenya are from the 2014 DHS;^3^ hence, the reference year for estimates included in Achoki and colleagues’ study is for some years before. Differences between predicted global health estimates and survey results can be substantial.^6^

Increasing global financial instability and competing non-health-related priorities necessitate continued investment strategies based on robust granular data platforms to ensure no person is left behind. Estimates of the burden of disease at subnational levels could inform targeted interventions, while evidence of risk factors could guide prioritisation of interventions. Without improvements to realising good quality data, we will be unable to understand and solve many of the world’s pressing problems. Efforts to improve collection of disaggregated data used to measure progress, strengthening of electronic data-capture systems and reporting rates, and ensuring all relevant data are gathered must be prioritised going forward. For public health, an ideal ambition must be that we no longer depend on modelled predictions of health but can measure it.
